# Treatment Default amongst Patients with Tuberculosis in Urban Morocco: Predicting and Explaining Default and Post-Default Sputum Smear and Drug Susceptibility Results

**DOI:** 10.1371/journal.pone.0093574

**Published:** 2014-04-03

**Authors:** Imad Cherkaoui, Radia Sabouni, Iraqi Ghali, Darya Kizub, Alexander C. Billioux, Kenza Bennani, Jamal Eddine Bourkadi, Abderrahmane Benmamoun, Ouafae Lahlou, Rajae El Aouad, Kelly E. Dooley

**Affiliations:** 1 Directorate of Epidemiology and Disease Control, Ministry of Health, Rabat, Morocco; 2 National Institute of Hygiene, Ministry of Health, Rabat, Morocco; 3 Moulay Youssef University Hospital, CHU Ibn Sina, Rabat, Morocco; 4 University of Washington School of Medicine, Seattle, Washington, United States of America; 5 Johns Hopkins University School of Medicine, Baltimore, Maryland, United States of America; 6 National TB Control Program, Directorate of Epidemiology and Disease Control, Ministry of Health, Rabat, Morocco; Universidad Nacional de La Plata, Argentina

## Abstract

**Setting:**

Public tuberculosis (TB) clinics in urban Morocco.

**Objective:**

Explore risk factors for TB treatment default and develop a prediction tool. Assess consequences of default, specifically risk for transmission or development of drug resistance.

**Design:**

Case-control study comparing patients who defaulted from TB treatment and patients who completed it using quantitative methods and open-ended questions. Results were interpreted in light of health professionals’ perspectives from a parallel study. A predictive model and simple tool to identify patients at high risk of default were developed. Sputum from cases with pulmonary TB was collected for smear and drug susceptibility testing.

**Results:**

91 cases and 186 controls enrolled. Independent risk factors for default included current smoking, retreatment, work interference with adherence, daily directly observed therapy, side effects, quick symptom resolution, and not knowing one’s treatment duration. Age >50 years, never smoking, and having friends who knew one’s diagnosis were protective. A simple scoring tool incorporating these factors was 82.4% sensitive and 87.6% specific for predicting default in this population. Clinicians and patients described additional contributors to default and suggested locally-relevant intervention targets. Among 89 cases with pulmonary TB, 71% had sputum that was smear positive for TB. Drug resistance was rare.

**Conclusion:**

The causes of default from TB treatment were explored through synthesis of qualitative and quantitative data from patients and health professionals. A scoring tool with high sensitivity and specificity to predict default was developed. Prospective evaluation of this tool coupled with targeted interventions based on our findings is warranted. Of note, the risk of TB transmission from patients who default treatment to others is likely to be high. The commonly-feared risk of drug resistance, though, may be low; a larger study is required to confirm these findings.

## Introduction

Tuberculosis (TB) remains a global health threat, with 8.6 million cases and 1.3 million TB-related deaths in 2012 [1]. One major obstacle to the control of TB is failure to complete the lengthy treatment of 6 or more months. Treatment default is defined by the World Health Organization (WHO) as treatment interruption of at least two months. Patients with pulmonary TB who default may have sputum samples that are smear positive for acid-fast bacilli (AFB), indicating high risk of transmission to others [2]. TB treatment default is also associated with an increased risk of mortality [3], [4].

Treatment default is complex and is influenced by patient, treatment, systems, and community-level factors that vary by setting [5]–[8]. Its effect on development of multi-drug resistant TB (MDR-TB) varies from 3–32% by country but is not well-understood, since most resistance surveillance programs only sample patients who return to care on their own rather than actively recovering patients for testing to avoid bias and because routine pre-treatment drug susceptibility testing (DST) is rarely done, making it impossible to determine if drug resistance existed before default or resulted from it [5]–[7]. To design effective programmatic interventions to address TB treatment default requires in-depth evaluations at the local level [9], [10]. In one interventional study, patients judged to be at high risk of default based on local risk factors received specialized services, and treatment completion improved markedly [11].

In Morocco, over 26,000 new cases of TB are reported annually, and the default rate nationally is about 3% [12]. TB incidence and treatment default, though, are greater in urban centers and can be as high as 400/100,000 and 10–15% in some areas. Patients who default are likely to have poor outcomes with retreatment and are at a high risk of defaulting again [13], [14]. The consequences of TB treatment default for drug resistance in urban Morocco have not been studied previously. Because the risk of resistance following default is assumed to be high, these patients are often treated with the same retreatment regimens as patients who relapse or fail treatment [8]. Retreatment regimens are more complex, toxic, and lengthy than standard first-line treatment.

In a previous qualitative study, we explored treatment default from the point of view of healthcare professionals who provide care to patients with TB in Morocco [15]. In this paper, we present results of a concurrent study of risk factors for TB treatment default based on patient interviews and compare these findings to responses given by healthcare providers. In addition, we describe post-default sputum and drug susceptibility results from patients with pulmonary TB so that the implications of treatment default can be understood. A simple survey tool that clinicians in Morocco can use to determine if their patient with tuberculosis is at high risk of treatment default is proposed.

## Study Population and Methods

### Study Sites

Morocco’s National Tuberculosis Program is well-established and funded by the Ministry of Health. TB care and medicines are provided free of charge. TB diagnosis, treatment initiation, and follow-up occur at regional public pulmonary clinics (CDTMR). Patients are given TB medications via Directly Observed Therapy (DOT) at local primary care clinics or dispensaries. Study sites included nine CDTMR and one referral hospital in cities with TB “hot spots” (TB incidence of ≥400/100 K): Tangier, Rabat, Salé, Casablanca, Kenitra, and Fez.

### Study Design

We conducted a questionnaire-based, case-control study between June, 2010, and October, 2011. Adult patients with definite or probable pulmonary or extrapulmonary TB who either defaulted from TB treatment (cases) or successfully completed it (controls) were enrolled. Treatment default was defined as an interruption in TB treatment for ≥2 consecutive months. Patients identified by review of the registries at study sites were contacted by clinic staff and asked to participate. Patients who defaulted and returned to clinic on their own were also enrolled. Upon enrollment of a case, the next two patients who presented for an end-of-treatment visit with an outcome of treatment success (treatment completion or cure) at that same site were enrolled as controls. To describe risk factors for default, a structured questionnaire was developed based on results of previous studies [5]–[8], [14]. Cases were also asked to describe in their own words the reasons they defaulted. Data collected via direct patient interview were augmented through chart review.

A blood sample was collected for HIV testing. A sputum sample was collected from cases for sputum smear evaluation according to the Ziehl-Nielson method. Samples were cultured on Lowenstein-Jensen media at the regional TB laboratory or the National TB Reference Laboratory (LNRT). Drug susceptibility testing (DST) for isoniazid (H), rifampin (R), ethambutol (E) and streptomycin (S) was performed on all positive cultures at LNRT as previously described [16]. Culture data from one city did not meet quality control standards and were excluded from final analyses. Study participants provided written informed consent. This study was approved by the Ethics Committee of the Mohammed V University Faculty of Medicine and Pharmacy of Rabat and by the institutional review board of Johns Hopkins University School of Medicine.

### Data Analysis

Using data from a previous retrospective study [14], we estimated that 80 cases and 160 controls would give us 90% power to detect a difference of 20% or more in the most important risk factors for default. To compare characteristics of cases and controls, we used Pearson’s χ^2^ or Fisher’s exact tests for categorical variables and student’s t tests for continuous variables. Multivariable logistic regression that included significant risk factors identified in univariate analyses was performed and used to develop a predictive model for treatment default. Variables with a p-value less than 0.2 in univariate analyses were included in the full model. Stepwise backward elimination methods were used to select the variables in the final model. For variables without evidence of multicollinearity, each variable’s significance as a predictor was tested by comparing the residual deviance of the nested model without the variable to that of the full model using the likelihood ratio test [17], [18]. Only those variables that were independently associated with default as indicated by a p-value less than or equal to 0.05 were retained in the final model. In addition, to avoid overfitting, Akaike’s Information Criterion (AIC) was taken into consideration in constructing the final model. In the model, knowledge of treatment duration was treated as a dichotomous variable. Those individuals who correctly stated the expected treatment duration for their TB disease were characterized as knowing treatment duration. Those who did not know or who gave a wrong answer were characterized as not knowing treatment duration. Smoking status was categorized as current, former, or never. In the model, current and never smoking were compared to former smoking. A survey tool to identify patients at high risk of default was developed by assigning points to each risk factor based on its coefficient in the predictive model. Different point cut-offs were tested to obtain the optimal sensitivity and specificity. Goodness of fit was tested using the Hosmer-Lemeshov test, where a p-value of >0.05 indicated that there was no significant difference between the collected data and that predicted by the model [19]. The models’ accuracy was tested by calculating the area under the receiver operator characteristic curve (AUC) and its 95% confidence interval (CI), where AUC that was significantly greater than 0.5 indicated that the model predicted the data better than chance [20]. Raw data were entered into Microsoft Access using EpiInfo. Data analyses were performed in SPSS (SPSS Statistics for Windows, Version 20.0. Armonk, NY: IBM Corp) and confirmed in R (Version 3.0.1, The R Foundation for Statistical Computing, Vienna, Austria).

For open-ended questions, the relative frequency of each type of response is presented along with representative quotes. Results of the quantitative analysis were compared to patients’ responses and to perspectives of local health care workers with extensive experience caring for patients with TB collected in a parallel study [15]. This mixed methods approach was used to explain and extend the results of the quantitative analysis [21], [22].

## Results

### Study Population

Our study enrolled 277 patients: 91 cases and 186 controls. Sixty-nine percent (69%) were male, 69% had salaries of <1800 dirhams/month, one-third finished primary school, and 30% were illiterate (Table 1). Twenty-nine percent (29%) were current tobacco smokers, and only 12% had ever drunk alcohol. Illicit drug use, mental illness, and comorbid chronic illness were rare. All patients were HIV-seronegative. Among the 91 cases, 65 (71%) defaulted after finishing the initial 2-month intensive phase of treatment. Time from default to return to TB care was 2 months for 22%, 3–4 months for 24%, 5–8 months for 25%, and ≥9 months for 29% of patients. Almost half (44%) of patients returned to clinic on their own; others returned after being contacted by phone (21%), following a home visit (22%), or after hospitalization (8%). The majority of patients knew the name of their disease, identified its respiratory route of transmission, and were aware of its potential lethality (Table 2).

**Table 1:** Comparison of characteristics of patients who defaulted from TB treatment (cases) versus patients who completed TB treatment (controls): results of univariate analyses.

Characteristic	Cases	Controls	p-value
	(N = 91)	(N = 186)	
**Sociodemographic characteristics**			
Age ≥50 years	6 (7)	36 (19)	<0.01
Male gender (n, %)	80 (88)	111 (60)	<0.001
Employment (n, %)			<0.01
Unemployed	29 (32)	77 (42)	
Student	0	11 (6)	
Housewife	0	6 (3)	
Part-time worker	18 (20)	22 (12)	
Full-time worker	42 (46)	64 (35)	
Salaried employee	2 (2)	5 (3)	
Income**			0.04
<1800 dirhams/month	70 (80)	122 (68)	
≥1800 dirhams/month	18 (20)	58 (32)	
Educational level			0.027
Unable to read	28 (31)	56 (30)	
Primary school	39 (43)	51 (28)	
Secondary school	21 (23)	61 (33)	
Attended university	3 (3)	17 (9)	
Number of people living with patient (mean, sd)	3.91 (3)	4.90 (3)	0.0044
Years at current address			0.61
<1	6 (7)	8 (4)	
1–3	9 (10)	15 (8)	
>3	76 (84)	163 (88)	
**Disease and treatment factors**			
Pulmonary TB	88 (97)	109 (59)	<0.001
Treatment type			<0.001
First treatment	53 (58)	172 (92)	
Retreatment	38 (42)	14 (8)	
Treatment initiation to symptom resolution:			0.001
<2 months	77 (85)	121 (65)	
≥2 months	14 (15)	65 (35)	
Treatment side effects			<0.01
None	65 (71)	113 (61)	
Mild	15 (17)	62 (33)	
Moderate	10 (11)	10 (5)	
Severe	1 (1)	1 (1)	
Treatment delivery			<0.001
Daily DOT	30 (33)	15 (8)	
No daily DOT	61 (67)	170 (92)	
HIV seronegative	91 (100)	184(100)	N/A
Diabetes mellitus	3 (3)	10 (5)	0.55
Mental illness	3 (3)	3 (2)	0.40
Current smoker	50 (55)	29 (16)	<0.001
Current alcohol use	21 (23)	13 (7)	<0.001
Current cannabis use	19 (21)	6 (3)	<0.001
**Access to care or social support**			
Can be reached by phone	46 (51)	96 (64)	0.046
Lives >5 km from clinic	14 (15)	20 (11)	0.27
Lives in rural area	13 (14)	28 (15)	0.84
Told about TB diagnosis			
Family	84 (92)	180 (97)	0.10
Friends	45 (50)	113 (61)	0.07
Colleagues	12 (13)	24 (13)	0.95
Work interference with treatment			<0.001
Work does not affect treatment	63(71)	179 (96)	
Work interferes with treatment due to:			
Long distance to clinic	4 (5)	1 (0.5)	
Long work hours	7 (8)	3 (2)	
Seasonal work	5 (6)	2 (1)	
Another reason	10 (11)	0	

**n = 268, 6 controls and 3 cases declined to respond to this question; median urban income in Morocco was 6,100 dirhams/month in 2009.

**Table 2:** Knowledge of patients about TB and its treatment.

Questions and responses	Cases	Controls	p-value
	(n, %) (N = 91)	(n, %) (N = 186)	
**What is the name of the disease for which you are being treated in this clinic?**			0.48
Tuberculosis	61 (67)	135 (73)	
Respiratory illness	23 (25)	35 (19)	
I don’t know	7 (8)	15 (8)	
**How long does TB treatment take to complete?**			<0.001
I don’t know	25 (28)	26 (14)	
6–9 months	58 (64)	159 (86)	
Other response	7 (8)	1 (0.5)	
**What causes TB?**			0.07
A microbe	25 (27)	61 (33)	
Cold weather	33 (36)	51 (27)	
Bad water, food, or hygiene	4 (4)	9 (5)	
I don’t know	14 (15)	49 (26)	
Other response	15 (16)	16 (9)	
**Can TB be transmitted from person to person? If so, how is it spread?**			0.31
Yes, by respiratory route	70 (77)	139 (75)	
Yes, by physical contact	5 (5)	8 (4)	
Yes, by sexual contact	3 (3)	1 (0.5)	
No, it is not spread from person to person	6 (7)	19 (10)	
I don’t know	7 (8)	19 (10)	
**What are the risks of stopping TB treatment early?**			
There is a risk that you will not be cured	56 (62)	130 (70)	0.16
There is a risk that the disease will be transmitted to others	26 (29)	56 (30)	0.79
Stopping treatment early may make the disease resistant/more difficult to treat	38 (42)	72 (39)	0.63
Stopping treatment early may result in complications	36 (40)	61 (33)	0.26
I don’t know/can’t provide any risks	7 (8)	14 (8)	0.96
**Can people die from TB?**			0.61
Yes	83 (91)	166 (89)	
No	8 (9)	20 (11)	

### Patient-reported Reasons for Default and Completing Treatment

According to the patient survey, the most commonly chosen reasons for default were resolution of symptoms (32%), side effects (14%) or “other” (32%). In open-ended questions, 17% said the reason for default was multifactorial, while 21% cited personal or family problems: *“I left for Khemisset because I lost my mother. I stayed there to deal with family problems.” “My father died.” “I am old and there was no one to help me get medications.” “I was in a traffic accident and had multiple fractures that prevented me from going to get medications.” “I had a problem with my husband. I lost my child.” “I had a fight with my father and left for Agadir.”* Others abandoned treatment because of symptom resolution, travel (12%), relocation for work (7%), or a combination of these: *“I felt well, so I thought I was cured.” “I got a job in Tangier and left.” “I felt well and did not think about my treatment because of alcohol.” “I stopped treatment after I moved. I didn’t know I could transfer my care.”* Other reasons for default included inability to take time off work (7%), incarceration (8%), and unwelcoming medical personnel (6%): *“I was in prison for 7 months.” “My work was hard and not compatible with treatment.” “Because of conflict with personnel at the primary care center.” “I had vomiting and stomach aches because of medications.”*

When asked what could have prevented default, common responses included more education about TB (12%), stable employment and more flexible work hours (11%), money or better living conditions (9%), resolution of conflicts with family (8%), help from family (4%), more welcoming medical personnel (4%), or nothing (11%). In survey questions to the 186 controls, the most common reasons for completing treatment were desire to be cured (93%), doctor’s advice to finish treatment (41%), not wanting to transmit TB to others (24%), and fear for one’s health (20%). In open-ended questions, patients emphasized their desire to be cured, advice from doctors, family support, and fear of complications: *“I wanted to be cured and my doctor told me to do it.” “Because of my mother.” “My educated children insisted that I do it.” “I wanted to be cured and my neighbors died of tuberculosis.” “I wanted to work and had ambitions.” “For my own health and for my children.”*

### Quantitative Analysis and Predictive Model

**Figure 1:**
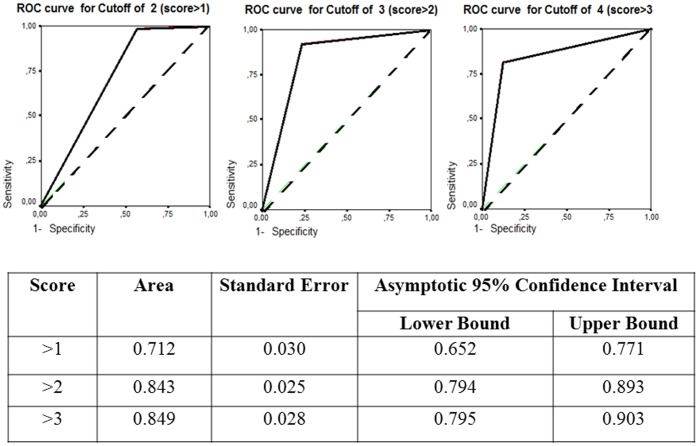
ROC curves for different point cut-offs for the proposed survey tool. The area under the ROC curve (AUC) for each score and its 95% confidence interval are provided.

In univariate and multivariate logistic regression analyses, default was associated with factors related to patient characteristics, knowledge about TB and its treatment, social support, and treatment organization (Tables 1–3). Age greater than 50 years, never smoking, and having shared one’s diagnosis with a friend were associated with treatment completion (Table 3). The final predictive model incorporated all of these factors and demonstrated good fit: the Hosmer-Lemeshov test was not significant (with χ^2^ = 3.1, p-value = 0.93). The AUC was 0.93 with 95% CI [0.90,0.96]. In the survey tool developed to identify patients at high risk of default, points were given for independent predictors of default: 2 points each for strong risk factors (logistic regression coefficient of >1.5) and 1 point each for moderate risk factors (Table 4). For never-smokers, one point was subtracted. This model had good fit and accuracy: the Hosmer-Lemeshov test was not significant with χ^2^ = 0.77, p-value = 1.00. The AUC was 0.85, 95% CI [0.80, 0.90] (Figure 1). A total score of 4 or more points had the highest AUC and was 82.4% sensitive and 87.6% specific for default. Of patients with a score of ≥4, 77% defaulted treatment; 91% of patients with a score of <4 completed treatment. Default rates were as follows: 1% for score <2, 10% for 2, 27% for 3, 52% for 4, 78% for 5, 92% for 6–7, and 100% for >7 points.

**Table 3:** Risk Factors associated with TB treatment default and level of significance in multivariable logistic regression model.

Characteristic	UOR*	95% CI	AOR**	95% CI	p-value
Knowledge of treatment duration	3.4	1.8–6.3	5.1	2.0–12.8	0.001
Retreatment	6.5	3.0–14.1	6.5	2.5–16.9	0.000
Current smoker	6.6	3.4–12.2	4.1	1.5–10.9	0.005
Has never smoked cigarettes	0.1	0.0–0.2	0.2	0.1–0.7	0.010
Work as hindrance to treatment	11.3	4.5–30.2	13.7	3.8–48.9	0.000
Age >50 years	0.3	0.1–0.8	0.1	0.0–0.4	0.001
Time from treatment initiation to resolution of clinical symptoms <2 months	2.9	1.5–5.9	4.4	1.7–11.4	0.003
Medicines provided daily via DOT	5.6	2.7–11.7	5.8	2.0–16.9	0.001
Having moderate or severe side effects from the TB treatment	2.2	0.8–5.7	7.3	1.8–29.7	0.006
Having told friend(s) about TB diagnosis	0.6	0.3–1.1	0.3	0.1–0.7	0.005

(*) Unadjusted odds ratio (univariate analysis).

(**) Adjusted odds ratio (multivariable analysis).

**Table 4:** Survey tool for determining risk of TB treatment default in urban Morocco.

Question	Point assignment	Points for this question
1. Are you younger than 50 years of age?	Yes = 2 points, No = 0 points	_____
2. Do you feel that work is interfering with your ability to take TB treatment?	Yes = 2 points, No = 0 points	_____
3. Are you taking a retreatment regimen for TB?	Yes = 2 points, No = 0 points	_____
4. Are you required to go to the clinic or dispensary daily to get your TB treatment?	Yes = 2 points, No = 0 points	_____
5. Do you or your doctor think you having moderate or severe side effectsfrom the TB treatment?	Yes = 2 points, No = 0 points	_____
6. Have you told any of your friends that you have TB?	No = 1 point, Yes = 0 points	_____
7. Are you a current smoker?	Yes = 1 point, No = 0 points	_____
8. Did your TB symptoms go away within 2 months of starting your TB treatment?	Yes = 1 point, No = 0 points	_____
9. Do you know how long your TB treatment is supposed to last?	No = 1 point, Yes = 0 points	_____
10. Have you ever smoked cigarettes?	No = −1 point, Yes = 0 points	_____
		TOTAL SCORE = ___

### Implications of Default from TB Treatment: Sputum Smear Positivity and Drug Resistance

Among the 91 cases, 53 (58%) had defaulted from an initial treatment regimen, and 38 (42%) had defaulted from retreatment. Thirty-eight (67%) patients had completed at least two months of treatment prior to default, with 31 (82%) having no missed doses by self-report and review of patient treatment cards. Eighty-nine (98%) had pulmonary TB; of these, 64 (72%) had sputum that was smear-positive for acid-fast bacilli (AFB). Of 54 evaluable cultures, 39 (72%) were positive for *Mycobacterium tuberculosis,* and DST was performed on all of these. Isolates from three patients, all of whom were on retreatment regimens at the time of default, were found to have drug resistance: one to isoniazid alone, the second to isoniazid and streptomycin, and the third to isoniazid and rifampin. No resistance was detected among isolates from patients defaulting from an initial treatment regimen. All patients with resistant isolates had completed the first two months of retreatment without missing any doses.

## Discussion

TB treatment default is an important public health problem that undermines TB control efforts. We explored factors that contribute to TB treatment default in urban Morocco using a mixed methods approach and information collected from both patients and health care professionals. We developed a simple scoring tool with high sensitivity and specificity for predicting default in this patient population. What are the implications of treatment default? In our study, 72% of patients with pulmonary TB who defaulted had a sputum sample that was smear positive for TB when they returned to care, indicating high risk of transmission to others; drug resistance, fortunately, was rare.

The goal of any epidemiologic study should be to propose concrete strategies for intervention and not simply to describe risk factors, or even independent predictors, of an undesirable clinical outcome. To support patients who are at risk of treatment default, it is important to understand the causal mechanisms behind the associations discovered through quantitative analysis [10]. It is helpful to place results within their societal and healthcare context. It is just as crucial to examine them through a lens aimed at understanding how people make decisions about their health in the context of their everyday lives. One validated theoretical framework for this is the information-motivation-behavioral skills theory (IMB), which has been helpful in predicting long-term treatment adherence in other settings [23]. In this framework, a patient’s ability to complete TB treatment depends on his knowledge about the disease, his motivation (a function of his attitude toward treatment, its costs and benefits, and encouragement/support from others), and skills or resources necessary to finish treatment, including confidence in one’s ability to take medications for a prolonged period of time [24].

Independent predictors of default from our quantitative analysis – younger age, retreatment, smoking, daily DOT, quick resolution of symptoms, work interference with treatment, treatment side effects, and friends not knowing about a patient’s TB diagnosis – can be classified into patient-related, structural, and treatment-related factors, and all have been associated with default in prior studies [6], [9], [25]–[28]. Patient-reported reasons for default often involved life problems that were of immediate concern and more salient to the patient than continuing TB treatment, including the need to continue to earn a living, conflict with family, death of a parent, or incarceration. Lack of knowledge about one’s ability to transfer care to another health center upon moving and confusion of symptom resolution with cure also were also very common.

In our study, most results of quantitative analysis, patient responses, and in-depth interviews with clinicians overlapped; rarely, they conflicted. Each method uncovered unique potential contributors to treatment default. We grouped risk factors for default and protective factors by source, as well as location within the IMB model (Table 5). The model points to possible causal mechanisms linking the risk factors and default. In a parallel qualitative study, doctors and nurses who provided care to patients with TB were aware of most of the risk factors found via quantitative analysis of patient questionnaire data and patient interviews, with the exception of personal and family problems. Clinicians also described health systems factors that were unknown to patients but were important contributors to default and delays in recovery of patients who had defaulted, including lack of time for patient education because of staff shortages, lack of appointment registers, and insufficient communication between pulmonary specialists and primary care providers [15].

**Table 5:** Organization of risk factors for default in urban Morocco within the information-motivation-behavioral (IMB) skills theoretical framework.

IMB	Univariate/multivariate* analysis	Patient responses	Medical personnel responses
**Information**	Not knowing treatment duration*	Lack of knowledge or understanding about treatment duration, what constitutes a cure, side-effects, ability to transfer care upon moving	Lack of knowledge or understanding about treatment duration, what constitutes a cure, side-effects, ability to transfer care upon moving or travel, lack of time for patient education because of staff shortages or low staff motivation, lack of public education campaigns about TB
**Motivation**	Daily DOT*, moderate/severe treatment side effects*, perception of work interfering with treatment*, quick resolution of symptoms*, no friends who know about TB diagnosis*, alcohol use, cannabis use, drug use, smoking	Personal or family problems, incarceration, unwelcoming clinic personnel, having to move for work, having to travel for personal reasons, resolution of symptoms, alcohol or cannabis use, fear of stigma, living far away from treatment site. Personal motivation to be cured, fear of complications, support from clinicians or family, concern about family or one’s health	Daily DOT, living far away from clinic, quick symptom resolution, treatment side-effects, interference with work, having to travel to find work or for personal reasons, cannabis, alcohol, drug use, mental illness, incarceration, unwelcoming clinic personnel, family support and involvement
**Barriers, Resources**	Low income, low level of education, age <50* (may be due to less life experience and less well-developed coping strategies at younger ages)	Low health literacy, lack of money for transportation, no income and need to make money despite illness, acute illness, no one to provide assistance with obtaining medications	Low education, low income and lack of money for transportation or inability to take time off work despite illness, lack of financial and staff resources to find patients who have defaulted treatment

*Statistically significant in multivariate analyses in this study.

Medical professionals frequently cited markers of low socio-economic status (SES), such as poverty, illiteracy, alcohol and drug use as important causes of default [15]. However, although these factors were associated with default in bivariate analysis, they were not mentioned by patients and not found to be independent predictors of default in multivariate regression. The association between low SES and poor health outcomes, including low treatment adherence and substance abuse is well documented in other disease settings. According to sociology studies, it may exist because people of low SES have a lack of economic opportunities, which often take precedence over treatment; may concentrate more on immediate problems instead of their health due to a bleak outlook on the future; may have latent traits that impact SES and health similarly, such as inadequate knowledge, risk-taking, and focus on short-term gain; may lack access to resources that could assist in treatment completion; often live in communities that exhibit less social cohesion and more negative peer influences; and may lack a sense of control over their lives that results in low self-confidence in finishing treatment [29]. Unlike low SES, both smoking and retreatment were found to be independent predictors of default in our study. Interestingly, we found that smoking was an independent predictor of both alcohol use and illicit drug use. Conversely, individuals who reported never smoking were rare among patients receiving retreatment. Like SES in other settings, it is possible that smoking is a marker for other social and behavioral factors that make default more likely. This merits further exploration.

Combining results from quantitative and qualitative analysis also provided insight into the possible mechanisms by which risk factors could result in default that were less intuitive, for example daily DOT. The utility of DOT is widely touted, and there is ample evidence that treatment completion is enhanced by DOT [30]. In our study, daily DOT was a predictor of treatment default. Many clinicians interviewed in a parallel study felt that daily DOT hindered treatment completion because it did not accommodate patients’ competing responsibilities (school, work, family) and because daily dispensing by staff reduced the time available for other components of good patient care. At some CDTMR, TB medications were given to patients every 7–14 days with default rates <5%. Instead of DOT, these sites focused on providing individualized care and improving health systems factors that contributed to default. More flexible patient-centered DOT strategies, including decentralized, community-based DOT; fewer DOT visits per week for patients with demonstrated adherence; and self-administration with adherence support are increasingly being tested, with good success [31]–[33]. Very few medical treatments are delivered in one-size-fits-all fashion, and the complexity and duration of TB treatment argue for increased flexibility in ensuring optimal adherence to treatment.

Default is commonly cited as a contributor to drug resistance. WHO guidelines recommend an 8-month retreatment regimen involving an injectable agent for the first two months for patients who default from initial treatment or second-line drugs for those at high risk of resistance. Prior studies have looked at resistance patterns only in patients who return to care on their own after defaulting treatment. Our study also included patients who were actively recovered by study clinicians. Among the patients from whom samples were sent for DST, the three individuals with drug-resistant TB were all on retreatment regimens at the time of default. None of the patients who had defaulted from an initial TB treatment subsequently developed drug-resistant TB. Because retreatment was an independent predictor of default in our study and drug resistance was rare, a larger prospective study of drug resistance that involves baseline, serial on-treatment, and post-default sputum sampling for DST is warranted to determine if the recommendation to treat patients who default from initial treatment with retreatment regimens should be reconsidered.

In several systematic reviews, simple interventions to improve treatment adherence by targeting a single risk factor related to patient characteristics–for example by providing monetary incentives to encourage clinic visits or general education about TB–have been ineffective or of only marginal benefit [34], [35]. On the other hand, strategies aimed at factors related to TB treatment organization, such as patient reminders or strategies for timely discovery and recovery of patients who default, have shown positive effects [36], including when practiced by clinicians at some of our study sites [15]. Patient-centered interventions that involve both patient-related and health-system determinants of default are most likely to be successful [11]. Such interventions will be most effective if they target patients at high risk of default rather than all patients, since they do not improve outcomes among patients already likely to complete treatment [37].

Few studies have used formal modeling to develop a scoring system to predict the probability of treatment default [38], [39]. By assigning points to independent predictors of default based on their logistic regression coefficients, we developed a scoring system that was 82.4% sensitive and 87.6% specific for TB treatment default in urban Morocco. Further work will be required to validate this tool, which must be coupled to assessments of interventions to be useful.

Our study has several limitations. We could only interview patients that we were able to locate, so our results may not be generalizable to all patients who default from TB treatment in urban Morocco. However, characteristics of participants in this study were very similar to characteristics of patients who default from TB treatment in Morocco in general [14], [15]; further the study team made a concerted effort to recover patients who had defaulted, rather than just enrolling patients who return to care on their own, to minimize potential bias. Culture results in one laboratory did not meet quality control standards, so we were unable to obtain reliable DST results from all cases. However, demographic and disease characteristics of patients for whom results were not available closely resembled the larger study population. Though our survey tool to identify patients at high risk of default had high sensitivity and specificity, it has not been validated. Prospectively testing a tool that has a high likelihood of identifying patients at high risk of default will require a thoughtful approach, as it must be married to interventional strategies to be ethical.

## Conclusions

TB treatment default is a complex public health problem that threatens TB control efforts. Our study explored TB treatment default in urban Morocco through a combination of qualitative and quantitative approaches that incorporated the views of patients and others involved in their care or its organization. Quantitative data were used to design a screening tool that may be used by local clinicians to identify patients at a high risk of default so they can benefit from targeted services to help them complete treatment. Qualitative data explained causes of treatment default more comprehensively and can be used to design relevant and realistic intervention strategies. Evaluation of optimal use of the screening tool and related interventions is warranted. While risk of TB transmission from patients who defaulted in our study was high, drug resistance was rare. A larger carefully designed study is needed to confirm our results and inform best practice for retreatment of patients who default.
